# Divergence times in demosponges (Porifera): first insights from new mitogenomes and the inclusion of fossils in a birth-death clock model

**DOI:** 10.1186/s12862-018-1230-1

**Published:** 2018-07-18

**Authors:** Astrid Schuster, Sergio Vargas, Ingrid S. Knapp, Shirley A. Pomponi, Robert J. Toonen, Dirk Erpenbeck, Gert Wörheide

**Affiliations:** 1 Department of Earth and Environmental Sciences, Palaeontology and Geobiology, Ludwig-Maximilians-Universität München, Richard-Wagner Str. 10, 80333 Munich, Germany; 2 Hawai‘i Institute of Marine Biology, 46-007 Lilipuna Road, Kāne‘ohe, Hawai‘i 96744 USA; 3 Harbor Branch Oceanographic Institute-Florida Atlantic University, 5600 U.S. 1 North, Ft Pierce, FL 34946 USA; 4 GeoBio-CenterLMU, Ludwig-Maximilians-Universität München, Richard-Wagner Str. 10, 80333 Munich, Germany; 5 SNSB - Bavarian State Collections of Palaeontology and Geology, Richard-Wagner Str. 10, 80333 Munich, Germany

**Keywords:** Mitochondrial genomes, Molecular clock, next-generation sequencing, Demospongiae, Fossilized birth-death model, Porifera, Molecular dating

## Abstract

**Background:**

Approximately 80% of all described extant sponge species belong to the class Demospongiae. Yet, despite their diversity and importance, accurate divergence times are still unknown for most demosponge clades. The estimation of demosponge divergence time is key to answering fundamental questions on the origin of Demospongiae, their diversification and historical biogeography. Molecular sequence data alone is not informative on an absolute time scale, and therefore needs to be “calibrated” with additional data such as fossils. Here, we calibrate the molecular data with the fossilized birth-death model, which compared to strict node dating, allows for the inclusion of young and old fossils in the analysis of divergence time. We use desma-bearing sponges, a diverse group of demosponges that form rigid skeletons and have a rich and continuous fossil record dating back to the Cambrian (~500 Ma), to date the demosponge radiation and constrain the timing of key evolutionary events, like the transition from marine to freshwater habitats. To infer a dated phylogeny of Demospongiae we assembled the mitochondrial genomes of six desma-bearing demosponges from reduced-representation genomic libraries. The total dataset included 33 complete demosponge mitochondrial genomes and 30 fossils.

**Results:**

Our study supports a Neoproterozoic origin of Demospongiae. Novel age estimates for the split of freshwater and marine sponges dating back to the Carboniferous and the previously assumed recent (~18 Ma) diversification of freshwater sponges is supported. Moreover, we provide detailed age estimates for a possible diversification of Tetractinellidae (~315 Ma), the Astrophorina (~240 Ma), the Spirophorina (~120 Ma) and the family Corallistidae (~188 Ma) all of which are considered as key groups for dating the Demospongiae due to their extraordinary rich and continuous fossil history.

**Conclusion:**

This study provides novel insights into the evolution of Demospongiae. Observed discrepancies of our dated phylogeny with their putative first fossil appearance dates are discussed for selected sponge groups. For instance, a Carboniferous origin of the order Tetractinellida seems to be too late, compared to their first appearance in the fossil record in the Middle Cambrian. This would imply that Paleozoic spicule forms are not homologous to post-Paleozoic forms.

**Electronic supplementary material:**

The online version of this article (10.1186/s12862-018-1230-1) contains supplementary material, which is available to authorized users.

## Background

The sequencing of sponge mitochondrial (mt) genomes greatly increased in the last decade [1–5]. Nevertheless, because some key taxa, such as Demospongiae, are still undersampled we are currently far from a representative number of mitochondrial genomes suitable to base molecular phylogenetic analyses at the level of orders and below. Presently, the species-poorest class Homoscleromorpha (106 species) has 14.2 % (15) mt genomes sequenced, whilst < 1 % have been sequenced for the other classes: Hexactinellida (679 species, 3 mt genomes), Calcarea (690 species, 1 mt genome), and Demospongiae (8225 species, 38 mt genomes) (Organelle Genome Resource database in GenBank; https://www.ncbi.nlm.nih.gov/genomes/OrganelleResource.cgi?taxid=6040). Therefore, there is a considerable need for denser taxonomic sequencing of mt genomes in sponges to allow for finer-scaled phylogenomic analyses.

Despite a few exceptions like *Poecillastra laminaris* (Tetractinellida: Astrophorina), where the mt genome was assembled using 454 pyrosequencing data [6], or the freshwater sponges *Spongilla lacustris* and *Ephydatia* cf. *muelleri*, which were assembled from Illumina (TruSeq) synthetic long-reads [7], all sponge mt genomes sequenced to date were assembled from Sanger sequencing reads (e.g. [8, 9]). However, Sanger sequencing is outdated regarding costs and yield, in particular if multiple mt genomes are pursued. Additionally, the use of this method can be challenging in demosponges due to the presence of extra protein-coding genes, long intergenic regions that may include repetitive sequences [4, 10], introns in the *cox1* gene [11, 12] and the existence of different gene arrangements [3]. An extreme example of the special characteristics of sponge mitochondrial genomes is the mt genome of *Clathrina clathrus* (Calcarea, Calcinea) which encodes 37 genes distributed in six linear chromosomes ranging 7.6-9.4 kb in size [13]. Despite their somewhat unique features, mt genomes have been successfully used to infer robust demosponge phylogenies [3, 8, 9], and gathering more sponge mt genomes will improve our understanding of the evolution of this animal group.

The demosponge order Tetractinellida comprises 23 families of world-wide distribution, of which 11 possess a rock-like skeleton built of interlocking spicules called desmas [14]. In contrast to most other demosponges, which fossil remains are usually limited to loose spicules (e.g. [15]), most tetractinellid families are known for their well preserved fossils and their continuous record (e.g. [14]). Among these are the Corallistidae of which characteristic desmas (dicranoclones) are known at least since the Late Jurassic with a continuous fossil record throughout the Mesozoic and Cenozoic [16]. However, among all tetractinellids, only three complete mt genomes (*Poecillastra laminaris* [6], *Geodia neptuni* [9] and *Cinachyrella kuekenthali* [8]) have been sequenced to date, none of which are from families of desma-bearing tetractinellids.

Sphaerocladina is another order of desma-bearing demosponges with a fossil record dating back to the Cambrian [17] from which no mt genome has been sequenced to date. However, this order is of particular importance for understanding demosponge evolution as it is regarded as the sister group to freshwater sponges [18–22], and thus constitutes a key taxon for reconstructing the last common ancestor of freshwater and marine sponges.

Given the rich fossil record of these rock-sponges (e.g. [14, 23–26]), sequencing the mt genomes of representatives of tetractinellids and Sphaerocladina will allow us in combination with the robustness of the phylogenies inferred from mt genomes, to provide a dated phylogeny of demosponges that can be used to better understand their evolutionary history.

Here, we generated size-selected reduced representation genomic libraries [27] to *de novo* sequence and assemble the mitochondrial genomes of six species of the orders Tetractinellida (mainly Corallistidae) and Sphaerocladina. Structural features of the six novel mt genomes are discussed. In total 35 demosponge mt genomes and 30 fossil taxa of diverse ages were used to infer a dated phylogeny of Demospongiae using the Fossilized Birth-Death (FBD) clock model. In contrast to the node calibrated molecular clock models, which only allow users to set the ‘oldest’ known fossil ages as constraints on certain nodes, the FBD model allows assignment of fossils of different ages to a clade without requiring morphological information about the fossils in the analysis [28]. Thus, the FBD model appears suitable for groups consisting of a rich and well studied fossil record such as desma-bearing demosponges (e.g. [23–26]). Until now the FBD model, in particular in the absence of a fossil character matrix, was used to estimate divergence times in bears [28], ferns [29], tetraodontiform fishes [30] and certain beeches [31], groups with fossils extending back to the Mesozoic. However, no attempt has been made to use this method to estimate the divergence time of groups, such as sponges, that radiated in the Early Paleozoic.

A dated phylogeny of Demospongiae using the Fossilized Birth-Death (FBD) clock model would be of value, because it would allow a comparison with previous molecular clock studies using different models and calibrations that suggested a Neoproterozoic origin of Demospongiae (see e.g. [32, 33]), as well as fossil discoveries from this age (e.g. [34]). Furthermore, being able to date the split of marine and freshwater sponges would allow for an understanding of freshwater sponge origin and their recent radiation (see e.g. [35, 36]). Dating the origin of Tetractinellida for the first time would allow to explain whether tetraxial-like spicules from the Middle Cambrian [37, 38] are homologous to those found in Recent tetractinellids. The dating of the suborders Spirophorina, Astrophorina and the family Corallistidae would allow for a comparative analysis of putative fossil appearance dates with our estimated divergence times. Together these data would provide a first step towards a better understanding of demosponge divergence and their origin.

## Methods

### DNA extraction and Illumina library preparation

Genomic DNA was extracted using a standard phenol-chloroform protocol [39] from frozen (-80°C) sponge tissue of five species (*Corallistes floreana* [40], *Corallistes* sp., *Neophrissospongia* sp., *Craniella wolfi* [40], *Vetulina stalactites* [41]), subsampled from the Harbor Branch Oceanographic Institute (HBOI; USA, Florida) collection, and one specimen of *Cinachyrella alloclada* collected freshly and preserved at -80°C. Detailed information on the samples used including museum vouchers, location, collection date and depths is provided in Additional file 1. DNA was purified with AmpureXP (Agentcourt) beads 3-5 times, according to the manufacturer's protocol, to remove degenerated DNA fragments and/or secondary metabolites. The samples were quantified using the AccuClear Ultra High Sensitivity dsDNA assay on a SpectraMax M2 plate reader (Molecular Devices, Sunnyvale, California). The libraries were prepared following the ezRAD method [42]. Two frequent cutter restriction enzymes, *MboI* and *Sau3AI* (New England BioLab) digested 1.0-1.3 μg DNA at the GATC cut site for 6 h at 37°C [27]. Digested products were cleaned with Ampure XP beads and eluted in 25 μl HPLC water. Then Illumina adapters were ligated on following the KAPA Hyper Prep Kit v1.14 (Wilmington, MA) guidelines with a modified size selection at 350-750 bp and library amplification [42]. Upon passing quality control steps (Bioanalyzer and quantitative real-time RT-PCR), all six libraries were 300 bp pair-end sequenced on an Illumina MiSeq (Illumina, Inc.) at the Hawai’i Institute of Marine Biology (HIMB) Genetics Core facility (Hawaii, USA).

### Mitochondrial genome assembly

Forward and reverse pair-end sequences (~2 million reads per library) were merged using the Paired-End reAd mergeR (PEAR) [43] software as implemented in our own Galaxy platform. A minimum overlap of 10 bp, a possible minimum length of the assembled sequences of 50 bp and a quality score threshold for the trimming of low quality parts (including adaptors and barcodes) of 20 was used. Paired sequences were imported in Geneious® v8.1.8 ([http://www.geneious.com, [44]) and a custom BLAST database for each library was built. For each library sequenced, one closely related mitogenome was downloaded from NCBI and used as a reference genome to map mt reads against and assemble the mt genomes (Additional file 1). The entire custom database was blasted against all reference genome protein, rRNA, and tRNA genes, as well as intergenic regions. To check for possible contamination, all reads were assembled separately and blasted against the NCBI database; non-sponge fragments, if any, were then excluded from the analysis. The remaining sponge sequences were mapped again to the reference genome. Possible intronic regions within the *cox1* of *Cinachyrella alloclada* were checked by blasting the library database against the *cox1*+intron region of *Cinachyrella alloclada* (HM032738). Consensus sequences were assembled *de novo* and mapped against the reference genomes respectively. Mitochondrial genomes were annotated using the similarity annotation tool (75%) and the ORF finder as implemented in Geneious®.

### Protein alignment and phylogenetic reconstruction

A concatenated alignment was built using Geneious® v8.1.8 from the 14 protein coding genes extracted from the mt genomes of 35 demosponge taxa. We used protein coding genes, as rDNA cannot be unambiguously aligned across the diverse taxa included in this analysis. Additionally, the translation into amino acids reduces noise. The final protein alignment was 3994 characters long, of which 1429 characters were constant, 285 characters were parsimony uninformative and 2280 characters were parsimony informative. This alignment was used to infer a Bayesian phylogenetic tree with PhyloBayes-MPI (v1.7) [45]. Two concurrent chains ran until convergence assessed using the *tracecomp* and *bpcomp* statistics in phylobayes, with the site-heterogeneous CAT-GTR model [46]. Burn-in was conservatively set to 30% of points sampled. Additionally, a Maximum Likelihood (ML) analysis with 1,000 bootstrap replicates was done using RAxML v8.0.26 [47] and the best-fitting evolutionary model (VT+Gamma+I+F) as suggested by ProtTest 3.4 [48]; the proportion of invariant sites parameter (I) was excluded as recommended from the RAxML manual [47]. We carried out both Bayesian and ML analysis to evaluate the effects of different models on the resulting tree topology and provided the summary tree in Additional file 2.

### Fossils and their assignments

The protein alignment was complemented by fossil taxa and their ages (Additional file 3 and https://github.com/PalMuc/mitoclocks2018 for the repository of all files used for analyses). As the FBD model requires the specification of point fossil ages [28], the youngest stratigraphic age for each fossil was taken (Additional file 4). In order to review the possible influence on the node ages with different parameters in BEAST, we carried out two different analyses, which differed by the following parameters: 1) number of fossils, to test for the sensitivity of fossil sampling density; 2) the origin of the FBD model and the root age; 3) the included/excluded Paleozoic fossils with sphaeroclone desmas because the homology of these spicules to the Mesozoic forms (see e.g. [49]) is debatable, and to assess the impact of removing the oldest fossil on the predicted ages (see Table 1). Fossils of 22 (BEAST analysis 1) and 30 (BEAST analysis 2) taxa belonging to five different demosponge orders (Poecilosclerida, Tethyida, Spongillida, Sphaerocladina and Tetractinellida) were extracted from the literature and linked to extant species or clades based on their suggested affinities to modern taxa (Additional file 4). These also include the oldest reliable fossils known to date from Poecilosclerida (*Ophiodesia* sp., 162 Ma [50]), freshwater sponges (Spongillida indet., 298 Ma [51]), Sphaerocladina (*Amplaspongia bulba*, 456 Ma [52], or *Mastosia wetzleri,* 155.5 Ma [53]) and Astrophorina (*Dicranoclonella schmidti,* 150.8 Ma [54]). Detailed information on all the fossils used, such as museum numbers, locality, stratigraphic level, taxonomic/systematic affinity to modern taxa, age range, references and Paleobiology Database (https://paleobiodb.org/#/) reference number are provided in Additional file 4. Fossil taxa were seen as either an ancestor or extinct sister taxa. Also because a representative demosponge morphological data matrix is difficult to compile due to e.g. the lack of microscleres in nearly all fossils, we placed them next to the appropriate subclades in the ML and BI trees (Additional file 3). Consequently, 10 defined higher taxa of both extant and fossil sponges were constrained based on the results of our BI analysis to be monophyletic, namely: Tetractinellida, Sphaerocladina, Poecilosclerida, Tethyida, Haplosclerida, Spongillida, Astrophorina, Spirophorina, Corallistidae and the yet unnamed clade combining Sphaerocladina and freshwater sponges (Additional file 4).

**Table 1:** Divergence time estimates (Ma) of demosponge clades of interest from two different analyses

	BEAST 2.4.3 parameters	
Nodes	Additional file 9 BEAST analysis 1 originFBD: 1000.0 root: 550.0 (Sperling et al., 2010 [59]) oldest Sphaerocladina fossil: 456.0 number of total fossils: 22 (Additional file 4, black colored text)	Figure 1 BEAST analysis 2 originFBD: 900.0 root: 515.0 (Botting, Cárdenas & Peel, 2015 [71]) oldest Sphaerocladina fossil: 155.5 number of total fossils: 30 (Additional file 4, black & red colored text)
A	28 (7, 53)	18 (5, 37)
B	483 (467, 517)	311 (298, 338)
C	166 (52, 304)	120 (40, 222)
D	391 (232, 564)	315 (216, 423)
E	185 (163, 246)	188 (155, 239)
F	279 (178, 396)	240 (173, 317)
R	875 (606, 1200)	594 (515, 730)

Estimates are given for the mean, and in brackets for the 95% highest posterior density interval

### FBD model settings

For both analyses the FBD model [28, 55], as implemented in BEAST v.2.4.3 [56], was used with an uncorrelated relaxed molecular clock model with default settings. No partitioning was applied on the data matrix as it had no influence on the divergence time estimation in (see Table 4 and Figure 3 in [37]). For the molecular sequence data a Gamma Site model with the JTT amino acid substitution model [57] was specified. As the start of the FBD process (root of the tree), based on previous molecular clock analyses [58, 59], we used two different ages (1000 Ma and 900 Ma) with a lognormal prior (mean=517 Ma, standard deviation min=471 Ma, max=624 Ma). Two hyperparameters were induced for the uncorrelated lognormal distribution (*ucldMean.c* and *ucldStdev.c*). As the substitution rates in Heteroscleromorpha mt genomes are considered to be low [3], we assumed an exponential prior distribution with 95% probability density on values <1 for the *ucldStdev.c* parameter. The diversification rate prior was set to an exponential with mean equal to 1.0 as the proportion of extant (33 species) and fossil taxa (22 or 30) used can be regarded as balanced. A beta distribution was chosen for the sampling proportion with Alpha 2.0. The default prior ‘uniform’ (0,1) was used for the turnover parameter (Additional file 4). Two independent Markov chain analyses were run for 400 million generations, sampling every 5000 generations. Runs were evaluated using Tracer v.1.6 [60] to assure stationarity of each Markov chain, an effective sample size (ESS) for all parameters over 200, and convergence of the independent runs. The first 25% of the sampled tree topologies from both analyses were discarded as burn-in, and the remaining trees were combined in LogCombiner and summarized in TreeAnnotator (both programs were implemented in the BEAST package) with mean divergence times and 95% highest posterior density (HPD). Before this, all fossils were removed from the tree using the FullToExtantTreeConverter tool (a tool implemented in BEAUti v.2.4.3). Possible prior influences to the posterior distribution estimates were checked by specifying the sampling from the prior only and rerunning the analysis. A summarized comparison of the turnover, diversification and sampling proportion of both runs and the priors is provided in Additional file 5, indicating that the number of fossils used are sufficient for our analyses.

Additionally, node ages of interest from both BEAST analyses (split of freshwater sponges and Sphaerocladina, Tetractinellida, Astrophorina, Spirophorina and Corallistidae) were extracted from the combined log-output-files (Additional file 6) and histograms showing the frequency distribution of the posterior age estimates were plotted in RStudio [61, 62], indicating the 95% highest posterior density interval (HPD), the means, and standard deviations (Additional file 7). A repository for all files used in this approach is available at https://github.com/PalMuc/mitoclocks2018

## Results and Discussion

### Mitochondrial genome organisation – a general comparison

While this approach has proven useful in other taxa such as molluscs and cnidarians [63, 64], here we provide the first complete mitochondrial genomes obtained from size-selected reduced representation genomic libraries of sponges. For all six libraries, we obtained more than 2 million reads of a minimum length of 50 bp and a quality score >20. All mitochondrial genomes were circular and vary in length and GC-content between 17,364 and 20,261 bp and 32.8% to 35.7% respectively (Additional file 8), which is in line with mitogenomes of other Heteroscleromorpha (see e.g. [3]). All mitogenomes contain 24 tRNA genes, 14 protein-coding genes and two ribosomal RNA genes and have the same gene order and coding strand as their reference genomes. The mitochondrial genome of *Cinachyrella alloclada* (GW3895) contains a 1,141 bp long group I intron in the *cox1* gene, which encodes for a homing endonuclease gene (HEG) of the LAGLIDADG family (Additional file 8). This intron is inserted at nucleotide position 723 with respect to the *cox1* sequence of *Amphimedon queenslandica* as previously found in several other species of the genus *Cinachyrella* (e.g. [11]). In *Corallistes* spp. and *Neophrissospongia* sp., four gene pairs overlapped (*atp8*/*atp6* (1bp), *nad4L*/*cox1* (13bp), *nad4*/*trnH*_(gug)_ (21bp), and *nad6*/*trnA*_(ugc)_ (10bp)) as previously reported for *Geodia neptuni* [9]. A further gene-pair overlap of 23 bp (*nad5*/*trnA*_(ucg)_) was located in *Vetulina stalactites* (Sphaerocladina), the same as found in freshwater sponges (e.g. *Eunapius subterraneus* and *Ephydatia muelleri*) [65]. Compared to the closest reference genome available to date (*E. subterraneus;* 88.5% pairwise sequence identity), *Vetulina stalactites* is 4,589 bp shorter (total 20,261 bp), shows reduced intergenic regions and lacks one tRNA gene (*trnR*_(ucg)_). The gene order and coding strands in *V. stalactites* is the same as for *E. subterraneus.* Although all freshwater sponges are known to possess various repeat motifs (direct, inverted and palindromes) in their mt genomes, some of which form repetitive hairpin structures (e.g. [4, 66]), none of these features were found in the mt genome of *Vetulina stalactites.* The same applies to other assembled mitogenomes despite their presence in other heteroscleromorphs (e.g. *Suberites domuncula* or *Axinella corrugata*, see Erpenbeck et al. [10]), which suggests that such repeat motifs evolved several times independently in sponges with large intergenic regions.

### Phylogenetic analyses

Our ML and BI trees corroborate the sister group relationship of the marine order Sphaerocladina (*Vetulina*), which is morphologically characterized by the possession of sphaeroclone desmas, to freshwater sponges (Spongillida) (Additional file 2), therefore supporting previous findings from ribosomal and partial mitochondrial single gene data [18–22]. Of the five Tetractinellida sequenced in this study, *Corallistes* spp. and *Neophrissospongia* sp. form a supported clade within the suborder Astrophorina. Furthermore, *Cinachyrella alloclada* is sister *to C. kuekenthali* and with *Craniella wolfi* forming a supported clade within the subclass Spirophorina (Additional file 2). This study increases the number of currently sequenced mt genomes within the order Tetractinellida by five and supports previous phylogenies of this order based on single genes (see e.g. [20, 22, 67–69]).

### Implications for divergence time estimates for Heteroscleromorpha

**Fig. 1:**
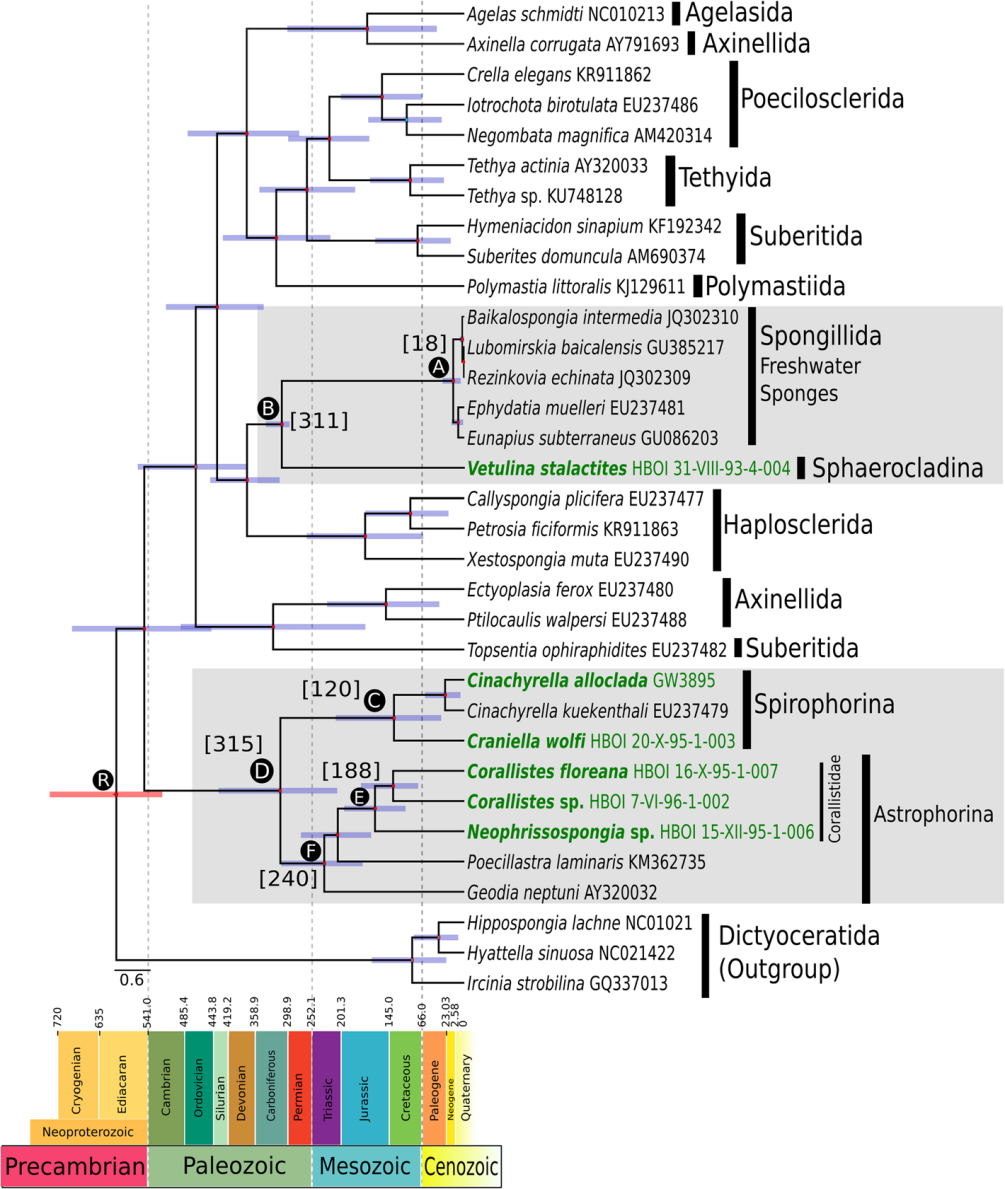
Time calibrated phylogeny of Demospongiae based on parameters of BEAST analysis 2 plotted on stratigraphic chart. New sequenced species are in dark green and bold. Taxonomic clades of interest are shaded in light gray. Error bars on node ages are in purple. Nodes of interest are marked with capital letters A-F on the nodes and correspond to node ages listed in Table 1. Numbers in brackets represents mean age estimations in Ma. The capital letter R specifies the root age of the dated phylogeny

The present study provides the first dated phylogeny of Heteroscleromorpha based on mt genomes and the relaxed molecular clock FBD model. The two analyses performed indicate a Neoproterozoic divergence of Heteroscleromorpha/Keratosa (node R, see Table 1, Fig. 1, Additional file 9). As the origin time of the FBD process should be greater than the maximum value of the root age with a log-normal distribution [28], we obtained different divergence times for Heteroscleromorpha/Keratosa in both analyses with the root age affecting the divergence time (see node R, Table 1, Fig. 1). Previous ages estimated for crown-group Demospongiae, using different software, clock-model settings, and taxon sets, varied between 657–872 Ma (e.g. [32, 37, 59, 70]), which is in the range of both of our analyses (see node R in Table 1, Fig. 1 and Additional file 9). The first reliable fossil representing crown-group Demospongiae was described by Botting et al. [71] from the early Cambrian (515 Ma). As this fossil only constitutes a minimum age it does not contradict a possible Neoproterozoic divergence of demosponges. This deep origin of crown-group Demospongiae concurs with the first appearance of demosponge-specific biomarkers (24-ipc sterol) in rocks dating 540-650 Ma (Neoproterozoic) and today present in all major demosponge clades [58, 72]. Although, we did not include any body fossil sponges as stem lineage due to uncertain assignments to modern groups, the discovery of a 600 Ma old body fossil, interpreted as a poriferan stem group descendant, provides additional paleontological evidence of an early sponge divergence [34].

### Inferred divergence scenarios for the split of marine and freshwater sponges

Many previously published molecular dating studies of Porifera are based on mitogenomic datasets, although hampered by incomplete taxon sampling, for example lacking freshwater and desma-bearing sponges. Consequently, inferences of divergence times for key demosponge taxa, such as the split between marine and freshwater sponges could not be addressed. Now, with the complete mitogenome of *Vetulina stalactites* (Sphaerocladina) available, this study represents the first dated phylogeny that suggests a likely time frame for the split of marine and freshwater sponges. Hypothesizing that the oldest fossil with sphaeroclone desmas from the Paleozoic (*Amplaspongia bulba*, Upper Ordovician ~456 Ma) [52] resembles species with the same desma types as those found in the Mesozoic, although larger in size and more heavily silicified (e.g. [14]), our analysis dates the split between marine and freshwater sponges (Table 1, Point B in Additional file 9) to the Early Ordovician (~483 Ma). Sponges with a massively silicified sphaeroclone desma skeleton are well known in the Paleozoic, and were common during the Late Ordovician, Middle Silurian and Late Devonian (see e.g. [14, 49, 73–75]). However, no sphaeroclone desmas are reported from the Carboniferous until the Middle Jurassic, which represents a ~200 Ma gap in the fossil record (e.g. [14]). Due to this long gap, it is debatable whether the Paleozoic sphaeroclone desmas are homologous to those found in the Mesozoic and Cenozoic [14, 76], and therefore suitable as fossil constraint. If Paleozoic sphaeroclone desmas are excluded from the analysis, the mean age of the split between marine and freshwater sponges dates back to the Carboniferous and is ~172 Ma younger (see node B in Fig. 1, Table 1). It has been suggested that the lack of a fossil sphaeroclone desmas during the Carboniferous until the Mid Jurassic is due to the Permian-Triassic boundary (PTB) mass extinction [49], which led to the reduction in size of sponge spicules, the disappearance of certain sponge groups [77], and to the habitat displacement of several sponge taxa from shallow neritic environments to deeper bathyal waters (see e.g. [78]). Maldonado et al. [79] proposed that the observed decline and turnover of the sponge fauna in the Mesozoic resulted from the reduction of silica in the oceans. This hypothesis is corroborated by the lack of sphaeroclone desmas found around and past the PTB mass extinction as well as the observed change from massive-large sphaeroclones in the Paleozoic to smaller and less silicified sphaeroclones in the Mesozoic.

### Inferred timing of extant freshwater sponge diversification

The occurrence of the earliest freshwater sponge fossil spicule is dated to the Permo-Carboniferous [51] and constitutes the first and only known fossil record of freshwater sponges from the Paleozoic. The radiation of recent freshwater sponges, however, is dated as much younger in both of our analyses (18.0-28.3 Ma, Paleogene, Table 1, node A). Therefore, our results question Schindler et al's [51] interpretation as Paleozoic spicules. Also Schultze [80] interpreted the findings of Schindler and coworkers [51] as either marine or marine influenced, which again challenges the interpretation of this oldest described freshwater sponge. In contrast, fossil freshwater sponges with intact gemmules (i.e. freshwater sponge-specific buds for asexual reproduction highly resistant to desiccation, freezing and anoxia (e.g. [81–83]) are well-known from the lower Cretaceous [84], thus supporting a diversification of Recent freshwater sponges before the Paleogene (66 Ma). Yet, Peterson and Butterfield [70] suggested a divergence of 7-10 Ma for Recent freshwater sponges using a node-calibrated relaxed molecular clock approach, whereupon the study of Schuster [85] indicates a Paleogene divergence. The Paleogene records of freshwater sponges are known to be more diverse than the Neogene records [86, 87].

Our analysis includes three freshwater species (*Baikalospongia intermedia, Lubomirskia baicalensis* and *Rezinkovia echinata*, all Lubomirskiidae), all of which are known to be endemic to Lake Baikal [35, 36]. Our dated phylogeny suggests that this clade diverges during the Early Pliocene (~3.4 Ma, Fig. 1, node A), which correlate to the known fossil record for this area (3.2-2.8 Ma) [35, 36]. As gemmules are known from the fossil record since the lower Cretaceous [84], and are present in the Recent spongillids *Ephydatia* and *Eunapius,* but absent from Lubomirskiidae (see discussion in [88]), our data is consistent with the hypothesis that the most recent common ancestor of Spongillida possessed gemmules, which were subsequently lost in several endemic lineages such as the Lake Baikal Lubomirskiidae (see discussion in [88]).

### Inferred divergence scenario of Tetractinellida, Spirophorina and Corallistidae

We estimated a mean origin age for Tetractinellida of 315 Ma (Late Carboniferous) (node D, Table 1, Fig. 1, BEAST analysis 2), with a normal frequency distribution on the node age (Additional file 7, BEAST analysis 2). Indeed, a Carboniferous origin is late for this group considering previous estimates which point to a Middle Cambrian (~514 Ma) origin of this clade in addition to the earliest tetraxial-like fossil spicules known from the Middle Cambrian (510-520 Ma) [37, 38]. Despite these Cambrian fossil discoveries, the molecular clock analyses of Sperling et al. [59] (~385 Ma) and Schuster [85] (~345.7 Ma) provide support for a post-Cambrian origin of this clade. These contradictory results may have different explanations. First, due to their massive and thicker size, the Cambrian tetractinellid (tetraxial-like) spicules may not be homologous to post-Paleozoic forms [14, 49]. Second, the presence of aster-like and monaxon spicules in several recent demosponge groups other than the Tetractinellida may lead to the erroneous interpretation of the Cambrian fossil spicules. Third, the high level of secondary losses of various spicule types, in particular microscleres within Astrophorina [22, 67, 89], hamper unambiguous interpretation of their homology.

The astrophorid family Corallistidae (node E, Table 1, Fig. 1), characterized by dicranoclone desmas, is here dated to ~188.7 Ma (Lower Jurassic). The node age shows a left-skewed distribution to younger ages (Additional file 7, BEAST analysis 2), which correlates with the current known fossil record from the late Jurassic to Recent [14, 26]. Additional support for a Jurassic origin of the included Recent tetractinellids is provided by a node-based calibrated single-gene phylogeny (*cox1*) of Schuster [85], who dated Corallistidae to ~155 Ma. The only known fossil representative of the genus *Neophrissospongia* is described from the Early Campanian of Poland [26], but our analysis indicates a deeper origin dating back to the Middle Jurassic (Fig. 1). As this family shows one of the richest and continuous fossil records among the included taxa, we tested this clade for sampling sensitivity of the FBD clock model by increasing the number of fossils by 50% (Additional file 4, BEAST analysis 2). This increase in fossil sampling neither influenced our results positively (by reducing the error bars for instance) nor negatively, which corroborates other findings of Heath et al. [28] and Grimm et al. [29]. The investigation of the divergence ages of this desma-bearing demosponge family strengthens the Jurassic origin of this clade and provides additional information on possible calibration constraints on further molecular clock approaches.

The tetractinellid suborder Spirophorina (node C, Table 1, Fig. 1, BEAST analysis 2) is dated to ~120 Ma (Late Cretaceous). The frequency distribution on the node age indicates a slightly right-shifted normal distribution towards younger ages (Additional file 7, BEAST analysis 2). A characteristic diagnostic feature for this group is the presence of sigmaspire (S- to C-shaped) microclere spicules. Kruse ([90]: Plate 24) described a C-shaped microsclere from the Middle Cambrian Daly and Georgina Basin (Northern Territory in Australia), which he associated to “orthocladine” sponges. Mehl-Janussen [91] suggested the occurrence of Spirophorina in the Early Paleozoic, with a possible Cambrian origin, however, these observations cannot be supported by any of our analyses. As sigma-like spicules are also present in other demosponge lineages like e.g. in Poecilosclerida and Desmacellida, the discovered C-shaped microsclere described in Kruse [90] might not be homologous to those of Spirophorina.

### Notes and caveats in estmating divergence times in sponges using mt genomes

The mitochondrial (mt) rate of evolution differ considerable within Porifera. Among all 4 classes, demosponges show a comparatively low mt evolutionary rate (see e.g. [5]). This low evolutionary rate has here been considered as advantageous for dating deep nodes in the phylogeny. For instance, such an approach would not be feasible for calcareous or hexactinellid sponges, where the rate of mt evolution is much higher (see e.g. [5]), or for a reconstruction including all four extant classes of Porifera. Even though differences in the mt rate of evolution are observed between poriferan classes, mutation rates within Demospongiae are similar (see e.g. [5]). Except for the order Dictyoceratida, none of the other demosponge groups have been found to possess accelerated rates of mt genome evolution (see e.g. [5]). Therefore, mutation rate heterogeneity is unlikely to affect our results.

Furthermore, adding more genes, e.g., from the nuclear genome, has been shown to not necessarily shift the divergence times, but maybe narrow the distributions (error bars) on the nodes (see e.g. [92], Figure 4.). However, more important factors to consider in a dated analyses are the number of fossils as calibration points, possible uncertainties in their date estimations, as well using secondary calibration points, which consequently will shift when changing the estimated divergence times. (see e.g. [93, 94]).

## Conclusion

Here we successfully assembled six complete mitogenomes of different demosponge taxa generated by a size-selected reduced representation genomic library. Integrating these data into a novel mitogenome alignment in tandem with a newly tested relaxed molecular clock approach based on the FBD model, we provide new insights into the evolution of selected Demospongiae. The Neoproterozoic origin of Demospongiae is confirmed. Furthermore, the origin and diversification of the Tetractinellida is dated to ~315 Ma, the suborders Astrophorina to ~240 Ma, the Spirophorina to ~120 Ma and the family Corallistidae to ~188 Ma. Furthermore, we discovered that increasing the fossil sampling by 50% within the Corallistidae made no differences and indicates that this approach is relatively insensitive to fossil sampling density, which corroborates with the findings of other studies [28, 29]. Nevertheless, our estimated divergence times of different higher tetractinellid taxa such as the Astrophorina or Corallistidae can be further used for inferring finer-scaled divergence time estimates to shed new light on e.g. the correlations of secondarily spicule losses to possible geochemical/geological historical events in the past.

The split of freshwater sponges and marine Sphaerocladina is dated to ~311 Ma, most of which correlate with the fossil record. Additionally, we confirmed previously assumed recent (~18 Ma) diversification of freshwater sponges. These results, and in particular the dated split of freshwater and marine sponges, can be used as a root age for further dated phylogenies on freshwater sponges in order to get a better picture of e.g. their historical biogeographical processes such as the radiation timing in different ancient lakes.

## Additional files

Additional file 1: Species list and ezRAD library information. (XLSX 52 kb)

Additional file 2: Bayesian Inference molecular phylogeny of the Demospongiae, based on 14 protein coding genes. Maximum likelihood topology is congruent. Clade support values are posterior (left) and bootstrap (right), above branch lengths. (PNG 437 kb)

Additional file 3: XML BEAST file including data matrix and parameter setup as described in Materials and Methods. (XML 291 kb)

Additional file 4: Detailed information on the fossils used. (DOCX 158 kb)

Additional file 5: Tracer statistics of turnover, diversification and sampling proportion of the two runs and the prior from BEAST analysis 2. (PNG 138 kb)

Additional file 6: BEAST analysis 2 output log file. (LOG 106 kb)

Additional file 7: Histograms showing the distribution of selected nodes from BEAST analyses 1 and 2. (PNG 327 kb)

Additional file 8: Mitochondrial genome structure with genome size, gene annotations, GC-content in blue and AT-content in green. (PNG 327 kb)

Additional file 9: Time calibrated phylogeny of BEAST analysis 1 plotted on stratigraphic chart. New sequenced species are in dark green and bold. Taxonomic clades of interest are shaded in light gray. Error bars on node ages are in dark turquoise. Nodes of interest are marked with a capital letters A-F on the nodes and correspond to node ages listed in Table 1. The capital letter R specifies the root age of the dated phylogeny. (PNG 913 kb)

## Abbreviations

BEAST: Bayesian evolutionary analysis sampling trees
BEAUTi: Bayesian evolutionary analysis utility
BLAST: Basic local alignment search tool
Cox1: Cytochrome oxidase subunit 1
DNA: Deoxyribonucleic acid
ESS: Effective sample size
FBD: Fossilized birth-death model
GTR: Generalised time-reversible
HBOI: Harbor Branch Oceanographic Institute
HIMB: Hawai‘i Institute of Marine Biology
HPD: Highest posterior density
HPLC: High-performance liquid chromatography
Ma: Million years (mega-annum)
ML: Maximum likelihood
Mt: Mitochondrial
NCBI: National center for biotechnology information
ORF: Open reading frame
PCR: Polymerase chain reaction
PEAR: Paired-End reAd mergeR
PTB: Permian-triassic boundary
RAD: Restriction site-associated DNA
rRNA: ribosomal ribonucleic acid
RT-PCR: Real time PCR
tRNA: Transfer ribonucleic acid
