# Supplementary figures and images for: Divergence times in demosponges (Porifera): first insights from new mitogenomes and the inclusion of fossils in a birth-death clock model
Source: BMC Evol Biol. 2018 Jul 18;18:114. doi: 10.1186/s12862-018-1230-1 (PMC6052604; doi:10.1186/s12862-018-1230-1)

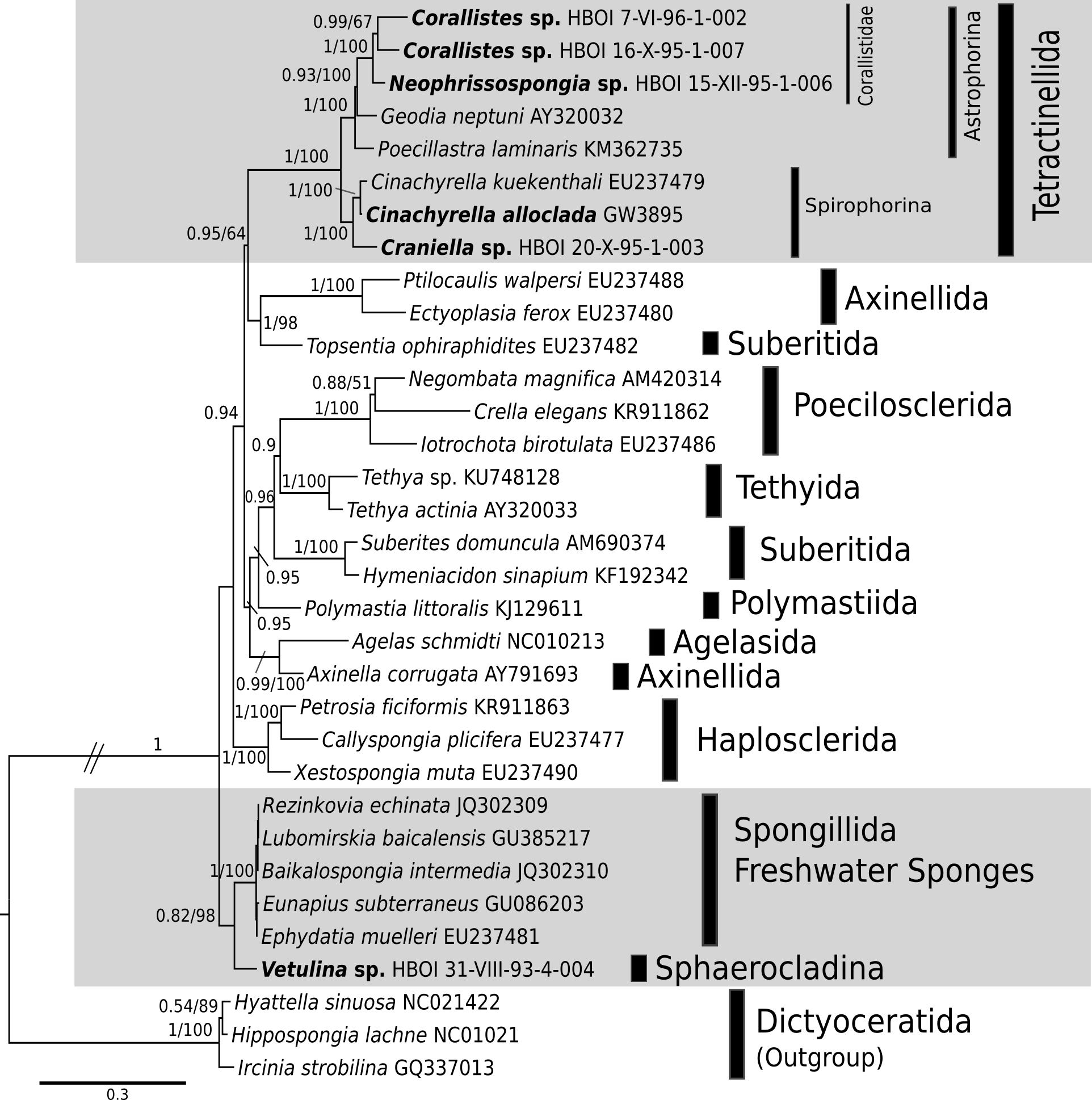

Supplement: Supplementary file 2 — Bayesian Inference molecular phylogeny of the Demospongiae, based on 14 protein coding genes. Maximum likelihood topology is congruent. Clade support values are posterior (left) and bootstrap (right), above branch lengths. (PNG 437 kb) [file 12862_2018_1230_MOESM2_ESM.png]

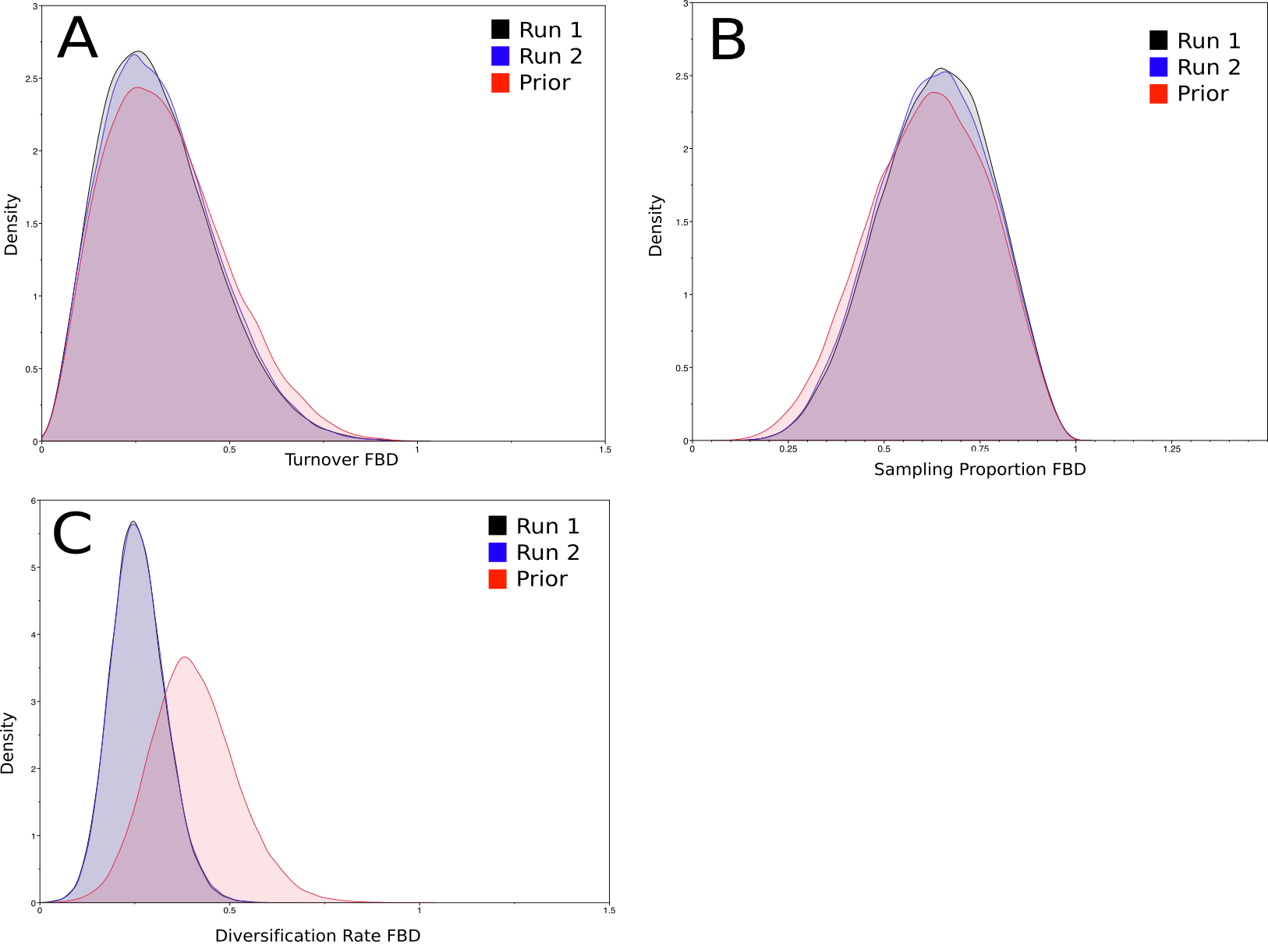

Supplement: Supplementary file 5 — Tracer statistics of turnover, diversification and sampling proportion of the two runs and the prior from BEAST analysis 2. (PNG 138 kb) [file 12862_2018_1230_MOESM5_ESM.png]

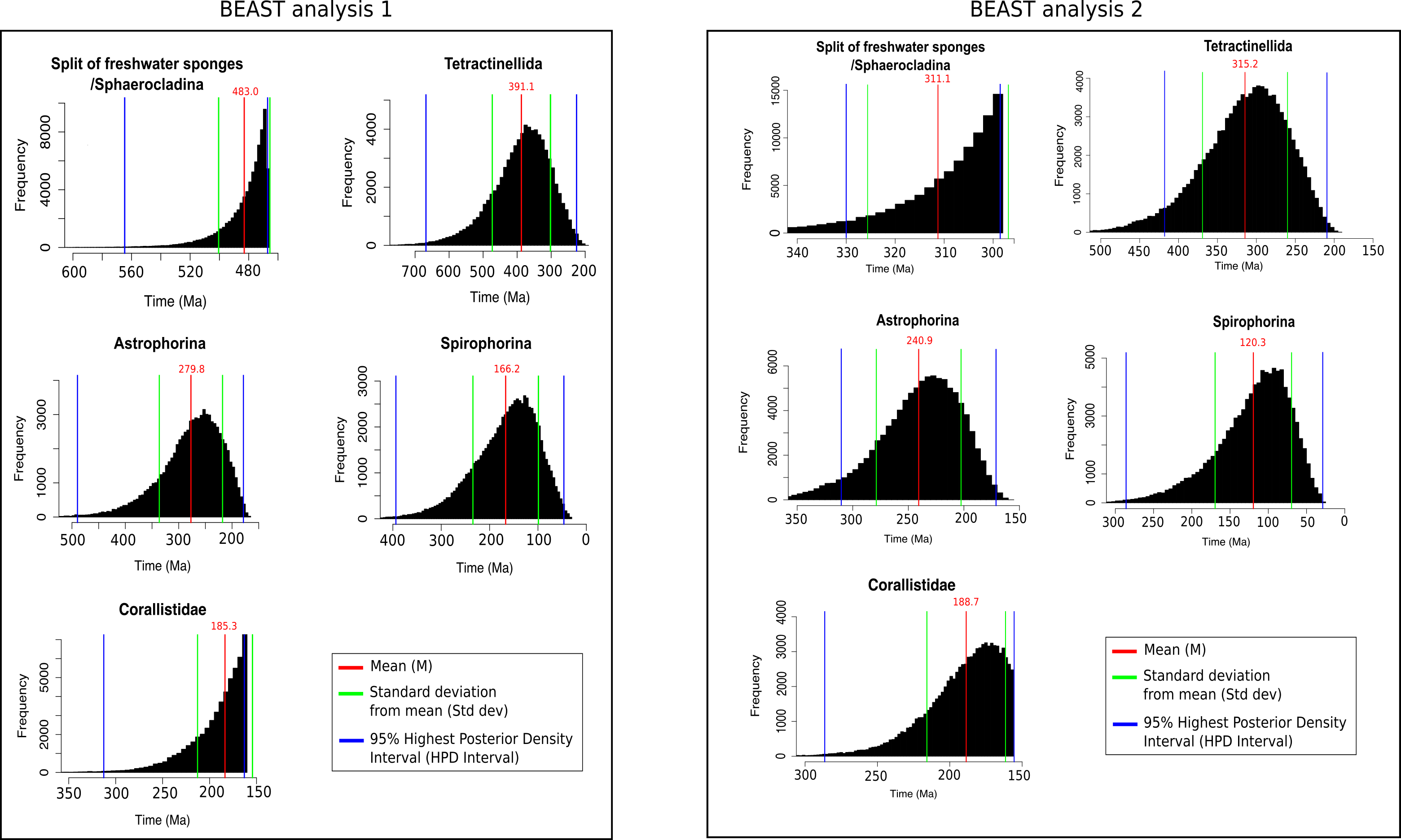

Supplement: Supplementary file 7 — Histograms showing the distribution of selected nodes from BEAST analyses 1 and 2. (PNG 327 kb) [file 12862_2018_1230_MOESM7_ESM.png]

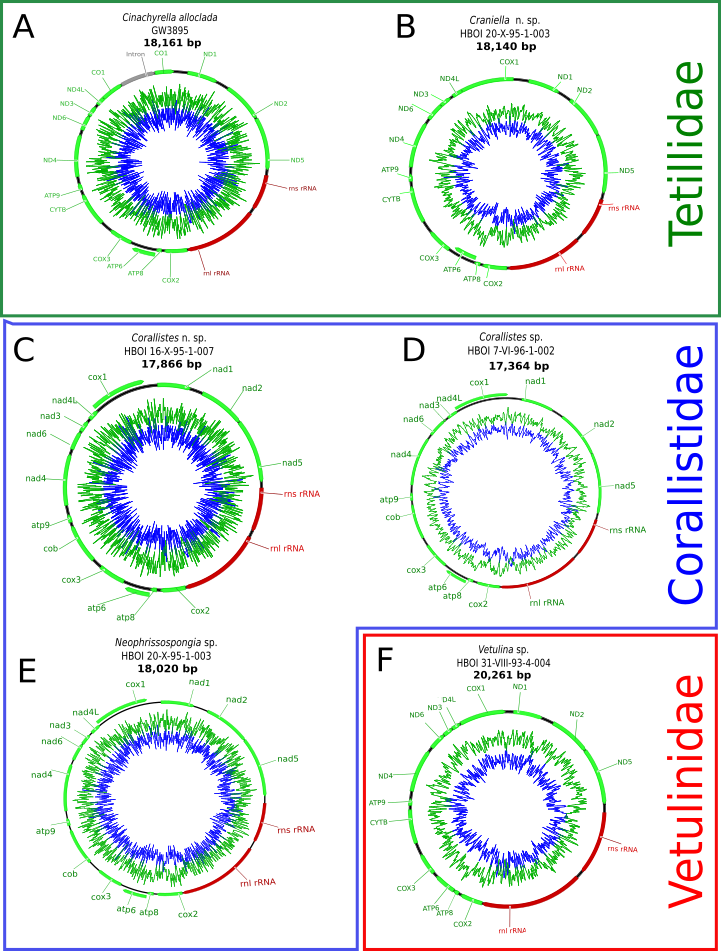

Supplement: Supplementary file 8 — Mitochondrial genome structure with genome size, gene annotations, GC-content in blue and AT-content in green. (PNG 327 kb) [file 12862_2018_1230_MOESM8_ESM.png]

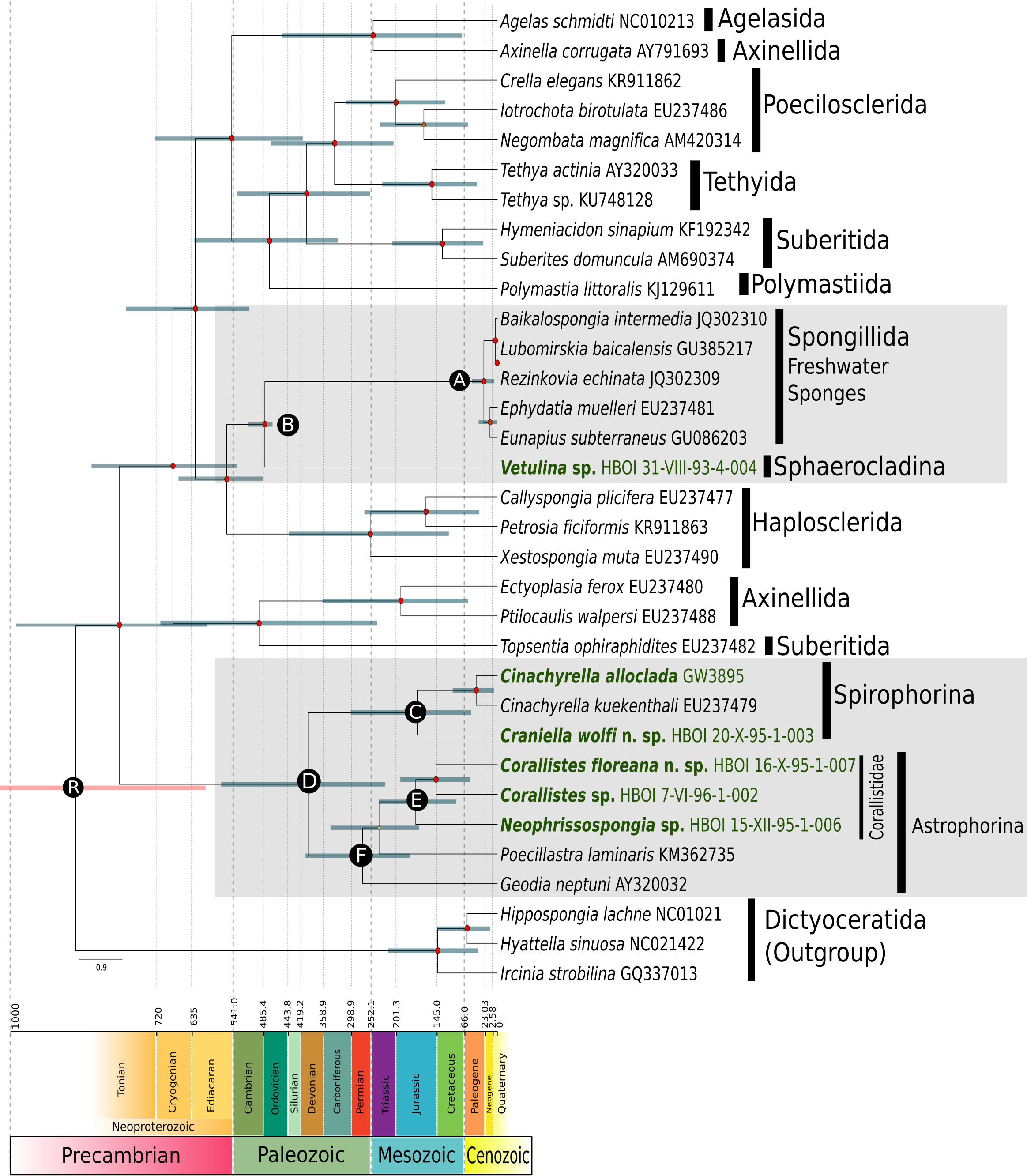

Supplement: Supplementary file 9 — Time calibrated phylogeny of BEAST analysis 1 plotted on stratigraphic chart. New sequenced species are in dark green and bold. Taxonomic clades of interest are shaded in light gray. Error bars on node ages are in dark turquoise. Nodes of interest are marked with a capital letters A-F on the nodes and correspond to node ages listed in Table 1. The capital letter R specifies the root age of the dated phylogeny. (PNG 913 kb) [file 12862_2018_1230_MOESM9_ESM.png]
