# Supplementary material for: Divergence times in demosponges (Porifera): first insights from new mitogenomes and the inclusion of fossils in a birth-death clock model
Source: BMC Evol Biol. 2018 Jul 18;18:114. doi: 10.1186/s12862-018-1230-1 (PMC6052604; doi:10.1186/s12862-018-1230-1)
Supplement: Supplementary file 4 — Detailed information on the fossils used. (DOCX 158 kb) [file 12862_2018_1230_MOESM4_ESM.docx]

| **Taxa** | **Ma (BEAST)** | **Stratigraphic range** | **Taxonomic/**  **systematic affinity to modern taxa after Morrow & Cárdenas 2015** | **Locality and stratigraphic level** | **Reference** | **Paleobiology Database reference number** |
| --- | --- | --- | --- | --- | --- | --- |
| Poecilosclerida |  |  |  |  |  |  |
| *Ophiodesia* sp. | 162.0 | Upper Jurassic | Poecilosclerida | Niwiska, Planula Zone, Polish Jura | J. Trammer 1989 | 2911 |
| *Crellastrina* sp. | 37.8 | Upper Eocene | *Crellastrina alecto* Topsent, 1898 | Southern part of W-Australia, Collection number ZPAL Pf.26 | Lukowiak 2016 | N/A |
| Tethyida |  |  |  |  |  |  |
| *Tethyastra* sp. | 37.8 | Upper Eocene | Tethyida | Blanche Point Formation, Fulf St. Vincent, Eastern part of South Australia | Lukowiak 2016 | N/A |
| Spongillida |  |  |  |  |  |  |
| *Ephydatia chileana* | 5,8 | Late Miocene | *Ephydatia* | Quillagua Formation, Cerro Mogote section, Chile, Atacama region, Holotype ZPAL Pf.12/1 | Pisera & Sáez 2003 | N/A |
| *Patomophloios canadensis* | 41.2 | Middle Eocene | Potamolepidae, Spongillida | Giraffe pipe, Lac de Gras, Canadian Shild Holotype ZPAL Pf. 23/A395 | Pisera et al. 2013 | N/A |
| *Paleospongilla chubutensis* | 100.0 | Cenomanian | *Spongilla alba* | Chubut Formation, Chubut River Vally, Patagonia/Argentine, paratype (Munich speciment) | Ott & Volkheimer 1972, Volkmer-Ribeiro & Reitner 1972 | N/A |
| *Eospongilla morrisonensis* | 145.0 | Upper Jurassic | Spongillida | Morrison Formation, Colorado, USA, Holotype USNM 496326 | Dunagan 1999 | N/A |
| Point 3, Spongillina indet. | 298 | Permo-Carboniferous | Spongillida | Lemberg/Saar-Nahe Basin, layer 4 Holotype PWL2004/5035a-LS | Schindler et al. 2008 | N/A |
| Sphaerocladina |  |  |  |  |  |  |
| *Mastosia wetzleri*,  Zittel 1878 | 155.5 | Kimmeridgian, Weiss Jura | *Vetulina* | Günzburg, Germany | Finks, Reid & Rigby 2004, Pisera & Lévi 2002 | N/A |
| *Pachytrachelus conicus*, Schrammen 1910 | 86.3 | Coniacian/Santonian | *Vetulina* | Sudmerberg and Petersberg, Germany | Schrammen 1910 | N/A |
| *Ozotrachelus conicus*, Roemer, 1841 | 41.2 | Early Lutetian, Eocene | *Vetulina* | Cengio dell'Orbo and Lovara quarries, Chiampo Formation, Veneto, Italy, Holotype: MCZ-PAL 3761 | Frisone et al. 2016 | 336158 |
| Point 2, *Amplaspongia bulba* | 456.0 | Upper Ordovician | Sphaerocladina | Coppermine Creek, Cliefden Caves, Malongulli Formation, Eastonian, New South Wales, Australia, | Rigby and Webby 1988 | 204288 |
| Tetractinellida |  |  |  |  |  |  |
| Spirophorina |  |  |  |  |  |  |
| *Paracinachyrella* sp. | 20.0 | 23.03-15.97 | *Cinachyrella* | Vienna Basin, Miocene of Slovakia, Cerova-Lieskove, Holotype: SNM Z21 | Lukowiak et al. 2014 | 307911 |
| Astrophorina |  |  |  |  |  |  |
| *Corallistes multiosculata* | 41.2 | Eocene, Lutetian | *Corallistes* | Cengio dell'Orbo quarry, Chiampo, Italy, volcaniclastics, Holotype: MSNVE–22912 | Frisone et al. 2016 | 336156 |
| *Procorallistes polymorphu*s, Schrammen 1901 | 83.6 | Campanian | Corallistidae | Oberg, Niedersachsen, Germany | Schrammen 1910 | 187343 |
| *Corallistes campanensis* | 83.6 | Lower Campanian | *Corallistes masoni* | NW of Belchatów lignite mine, Poland  Holotype: ULXXV/1D/1 | Swierczewska-Gladysz (2017) | no infromation |
| *Corallistes* sp. | 41.2 | Eocene, Lutetian | *Corallistes* | Cengio dell'Orbo quarry, Chiampo, Italy, volcaniclastics, Holotype: MCZ-PAL 3707 | Frisone et al. 2016 | 3062 |
| *Phrissospongia hoffmanni* (Wagner 1963) | 89.8 | Turonian/Coniacian | Corallistidae | Coastal sandstone, Neuburger Weiß Formation, Germany | Wagner (1963) | 194764 |
| *Phrissospongia glandiformis* | 83.6 | Santonian | *Neophrissospongia* | Near Naint Cyr, France | Moret 1926 | no information |
| *Neophrissospongia kacperskii* | 83.6 | Lower Campanian | *Neophrissospongia radjae* | NW of Belchatów lignite mine, Poland  Holotype: ULXXV/3D/1 | Swierczewska-Gladysz (2017) | no infromation |
| *Leiocarenus planus* | 155.5 | 155.7-150.8 | *Corallistes* | Hochwang, Germany, Upper White Jurassic Gamma and White Jurassic Delta, Reef 2758 | Pisera 1997 | 195277 |
| *Geodia hopetouni* | 37.8 | Upper Eocene | *Geodia* | Pallinup Formation, Fitzgerald Member, Southern Australia, Hamersley River section, Holotype: WAM 15.403 | Lukowiak & Pisera 2016 | no information |
| *Geodia* sp. | 83.6 | Lower Campanian | *Geodia* | Marls of the lowever Camanian in Zbyczyce, Poland, Z. Pal. UL Sp. II/729B | Hurcewicz 1966 | no information |
| *Gelasinophorus reitemeyeri* Schrammen 1924 | 86.3 | Coniacian | Corallistidae | Northern Germany | Schrammen (1924) | No information |
| *Spinocladia tubulata* | 112.6 | Aptian | Corallistidae | Can Casanyas Castellet, Coastal Sandstone in Spain | Lagneau-Herenger (1962) | 3291 |
| *Pycnoclonella dactyliformis* | 112.6 | Aptian | Corallistidae | Can Casanyas Castellet, Coastal Sandstone in Spain | Lagneau-Herenger (1962) | 191513 |
| *Schrammeniella bifurcata* | 83.6 | 83.6 | Lower (Late) Campanian | e.g. Belchatów and Zbyczyce, Poland | Swierczewska-Gladysz (2017) & Hurcewicz (1966) |  |
| *Schrammeniella scytaliforme*  Schrammen 1910 | 83.6 | Lower (Late) Campanian | Corallistidae | Offshore shelf marl/limestone  Teutonia Nord quarry, Misburg, Germany,  Belchatów, Pniaki, Zbyczyce, Skrajniwwa of Poland | Schrammen (1910)  Swierczewska-Gladysz (2017) & Hurcewicz (1966) | e.g. 187346 |
| *Pachinion canaliculatum* | 83.6 | Lower Campanian | Corallistidae | Marly opoka from Pniaki, Poland  Holotype: ULII/1614 | Swierczewska-Gladysz  (2017) | No information |
| *Pachinion scriptum Roemer 1864* | 83.6 | Lower Campanian | Corallistidae | Lelów area, Skrajniwa, Poland  Holotype: ULII/1641 | Swierczewska-Gladysz  (2017) | No information |
| *Pachinion scriptum*, Roemer 1864 | 84.9 | 84.9-70.6 | Corallistidae | Niedersachsen, Germany IV quarry | Schrammen 1910 | 187340 |
| Point 1, *Dicranoclonella schmidti* | 150.8 | 155.7-150.8 | Corallistidae | White Jurassic Zeta 2, Gussenstadt, Swabian Alb, Germany | Pisera 1997 | 195276 |
